# Does Change in Social Asymmetry Predict Cognitive Functioning and Dementia Risk Across Adulthood?

**DOI:** 10.1093/geroni/igaf122.1682

**Published:** 2025-12-31

**Authors:** Karina Van Bogart, Emorie Beck, Tomiko Yoneda, Kathryn Jackson, Sijing Shao, Anthony Ong, Eileen Graham

**Affiliations:** Northwestern University, Chicago, Illinois, United States; University of California, Davis, Davis, California, United States; University of California Davis, Davis, California, United States; Northwestern University, Evanston, Illinois, United States; FIU, Miami, Florida, United States; Cornell University, Ithaca, New York, United States; Northwestern University, Evanston, Illinois, United States

## Abstract

Abundant evidence exists on the separate links between loneliness, social isolation, and cognitive decline. However, little work has investigated the relative contribution of loneliness in relation to social isolation on cognitive decline across the adult lifespan. Understanding how change in this relative contribution (i.e., social asymmetry) relates to cognitive decline and dementia risk may shed light on differences in susceptibility to loneliness and key timepoints across the lifespan for intervention. In the current research, we use coordinated data analysis (CDA) incorporating several existing longitudinal samples across 21 different countries (ages ∼18-95) to examine how trajectories of social asymmetry relate to cognitive functioning and dementia risk across the lifespan using growth curve modeling. We examine how change in social asymmetry relates to change in cognitive functioning, and how change in social asymmetry relates to odds of dementia diagnosis at long-term follow-up. Preliminary results from one sample show a significant association between social asymmetry and cognitive functioning at longitudinal follow-up. Additional models will be expanded to assess bivariate change in social asymmetry and cognitive functioning in multiple samples. We will highlight the extent of replicability and generalizability of our findings, and discuss challenges and considerations of using CDA to longitudinally explore the novel index of social asymmetry.

